# Lateral Ridge Augmentation Using Low-dosage Bone Morphogenetic Protein-2 Functionalised Calcium Phosphate Cement

**DOI:** 10.1016/j.identj.2026.109545

**Published:** 2026-04-25

**Authors:** Lingfei Wei, Yuanyuan Sun, Yiqun Wu, Zhonghao Liu, Yuelian Liu

**Affiliations:** a Department of Oral Implantology, The Affiliated Yantai Stomatological Hospital, Binzhou Medical University, Yantai, China; b Department of Second Dental Center, Shanghai Ninth People's Hospital, School of Medicine; College of Stomatology, Shanghai Jiao Tong University, National Center for Stomatology; National Clinical Research Center for Oral Diseases; Shanghai Key Laboratory of Stomatology, Shanghai, China; c Department of Oral Cell Biology, Academic Centre for Dentistry Amsterdam (ACTA), Vrije Universiteit Amsterdam and University of Amsterdam, Amsterdam, the Netherlands

**Keywords:** Bone Morphogenetic Protein-2, Lateral ridge augmentation, Implant, Slow release, Bone regeneration

## Abstract

**Aims:**

To evaluate bone regeneration and osseointegration using low-dosage *Escherichia coli*-derived recombinant human bone morphogenetic protein-2 (ErhBMP-2) incorporated with calcium phosphate cement (CPC) via a biomimetic coating method during simultaneous lateral ridge augmentation with implant placement.

**Materials and methods:**

Chronic horizontal alveolar bone defects were created in the mandibles of 5 dogs. Before implant placement, defects were randomly assigned to different grafting groups: autologous bone, CPC, high-dosage ErhBMP-2 (1088 µg/g) adsorbed to CPC (ErhBMP-2 Ads. CPC), and low-dosage ErhBMP-2 (239 µg/g) incorporated with CPC (ErhBMP-2 Inc. CPC). Lateral ridge augmentation was performed at the buccal aspect of each defect during implant surgery. Bone regeneration and contour maintenance were assessed using micro-CT and histology 3 months postoperatively.

**Results:**

Micro-CT showed that the ErhBMP-2 Inc. CPC group developed substantial mineralised tissue covering the implant surface, with no exposed threads at the implant neck. Trabecular thickness in this group was significantly greater than in the CPC and autologous bone groups. Histology confirmed greater bone marrow formation and horizontal augmentation below the implant shoulder in the ErhBMP-2 Inc. CPC group compared with CPC alone. Collectively, the histological and radiographic findings were consistent with enhanced peri-implant bone regeneration in the ErhBMP-2 Inc. CPC group.

**Conclusions:**

ErhBMP-2 Inc. CPC enhanced peri-implant bone regeneration than CPC alone.

**Clinical relevance:**

Low-dosage ErhBMP-2 incorporated into CPC may offer a more predictable and effective approach for simultaneous lateral ridge augmentation and implant placement by enhancing bone regeneration and maintaining ridge contours.

## Graphical abstract

In a canine horizontal ridge defect model, low-dosage ErhBMP-2 incorporated into CPC enabled sustained growth factor release during simultaneous implant placement and lateral ridge augmentation. This approach enhanced osteogenesis, increased trabecular thickness, and preserved peri-implant ridge contours more effectively than CPC alone. Comparable bone regeneration was achieved with a reduced BMP-2 dose, highlighting its potential for safer and more predictable clinical translation.

**Figure:**
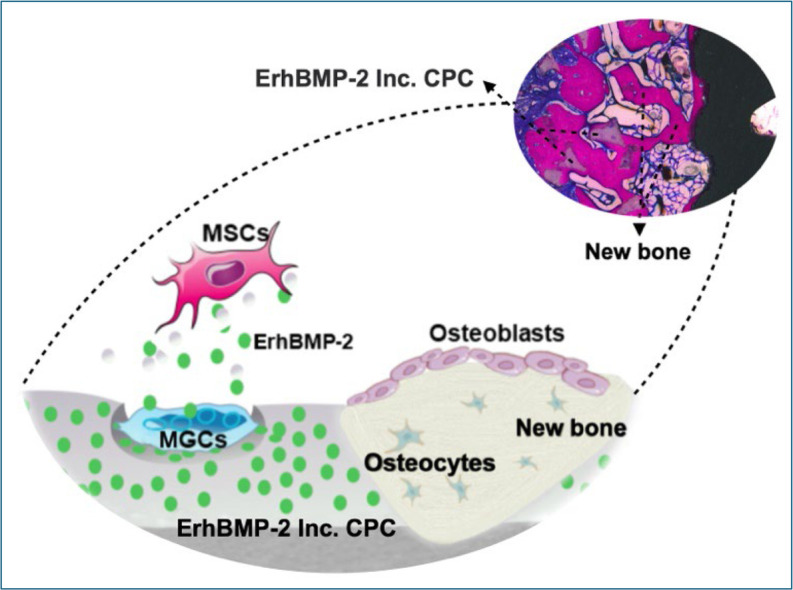

## Introduction

Lateral ridge augmentation is a pivotal technique for addressing bone defects in periodontal and peri-implant alveolar horizontal bone deficiencies. The process heavily relies on bone grafts and barrier membranes to facilitate successful bone regeneration.^1^, ^2^, ^3^ Various calcium phosphate-based bone grafts and substitute materials, including autogenous bone grafts, allogenic bone grafts, xenogeneic bone grafts, and synthetic calcium phosphate materials with or without additional components (such as growth factors, bioactive molecules, and therapeutic elements), are employed in dentistry.^4^, ^5^, ^6^

Although autogenous bone grafts remain the gold standard for bone regeneration, their use is limited by the need for an additional donor site and frequent donor-site complications.^7^ Allografts and xenografts serve as viable alternatives but present challenges such as potential disease transmission, immune reactions, and variable degradation rates.^8^ Deproteinised bovine bone mineral (DBBM), a chemically purified xenograft, is widely used with proven regenerative potential. However, DBBM exhibits slow degradation and remodelling,^9^ which has both advantages and drawbacks. Its slow resorption supports structural stability and long-term volume maintenance,^10^ but the low resorptive capacity leaves residual graft particles at the implant site,^11^ resulting in a bone structure different from autogenous bone.^12^

Synthetic calcium phosphates, such as hydroxyapatite (HA), tricalcium phosphate (TCP), biphasic calcium phosphate (BCP), and calcium phosphate cement (CPC), are extensively utilised in clinical practice due to their biocompatibility, osteoconductivity, rapid synthesis, and adaptability for different morphologies and particle size.^13^, ^14^, ^15^, ^16^ Nevertheless, the absence of osteoinductivity limits the applicability of these calcium phosphate-based materials in cases of severe bone defects.^17^

Bone morphogenetic protein 2 (BMP-2), a member of the transforming growth factor beta (TGF-β) superfamily, was approved by the Food and Drug Administration (FDA) in 2007 as a bone graft substitute for oral and maxillofacial reconstruction. With both osteoconductive and osteoinductive abilities, BMP-2 promotes bone formation by stimulating osteoblast proliferation and mesenchymal stem cell differentiation. However, its efficacy depends on the carrier and delivery system. The FDA-approved high concentration (1500 µg/mL) has raised concerns over dose-related complications, including inflammation, ectopic ossification, osteolysis, and wound issues.^18^ Because of its dose-dependent activity, reducing side effects without lowering efficacy is difficult.^19^ Therefore, developing safe, controlled, and sustained BMP-2 delivery systems has become a major research focus.

CPC is widely used as a bone graft substitute in clinical surgery due to its excellent drug-loading capacity and ability to deliver biological molecules.^20^ CPC can adsorb BMP-2 at a concentration of 1000 µg/g within its porous structure and release it into bone defect sites in vivo. Moreover, BMP-2-CPC has been shown to modulate macrophage polarisation, enhancing its immunoregulatory effects and promoting strong osteogenic potential.^21^

Our research team has pioneered an innovative approach that integrates low-dose rhBMP-2 (239 µg/g) into a biomimetic calcium phosphate coating on a CPC core. This method enables localised, controlled, and sustained BMP-2 release through a cell-mediated mechanism, mirroring the principles of natural bone remodeling.^22^^,^^23^ In comparison to traditional BMP-2 loading methods, our novel approach achieves similar efficacy with a significantly reduced BMP-2 dosage, thereby reducing the risk of side effects, such as inflammation, ectopic ossification, and swelling, which are associated with high-dosage BMP-2 application. Previous studies have demonstrated the efficacy of low-dose BMP-2 incorporated into BioCaP not only in vitro and in vivo^24^, ^25^, ^26^, ^27^ but also in clinical research.^28^^,^^29^ Although previous studies by our group and others have demonstrated the efficacy of low-dose BMP-2 delivered through biomimetic calcium phosphate systems in socket preservation and vertical ridge augmentation models, these experimental settings differ substantially from simultaneous lateral ridge augmentation with implant placement. Horizontal defects are characterised by buccal plate deficiency and limited lateral containment, which may compromise space maintenance and vascularisation. Moreover, immediate implant placement introduces additional mechanical and biological complexity, as bone regeneration must occur in close proximity to the implant surface while preserving ridge contour. Therefore, whether low-dose BMP-2 incorporation can maintain efficacy under these more demanding conditions remains to be determined.

In this study, we employed a novel bone graft substitute, involving a CPC core with low-dosage *Escherichia coli*-derived recombinant human bone morphogenetic protein-2 (ErhBMP-2) (239 µg/g) incorporated through a biomimetic coating method (ErhBMP-2 Inc. CPC), in lateral ridge augmentation surgery using a chronic horizontal alveolar bone defect model in dogs. Our aim was to assess the impact of ErhBMP-2 Inc. CPC on bone regeneration and alveolar contour maintenance around implants through radiographic and histomorphometry analyses.

## Materials and methods

In compliance with the modified ARRIVE guidelines for preclinical research, this process adhered to the "3Rs principles." All animal experiments were approved by the Ethical Review Committee for Animal Experiments at Weihai Desheng Technology Testing Co. Ltd. (acceptance number SQR-MD-DW-7.5-142-A/0-0701).

### Preparation of the ErhBMP-2 Inc. CPC

The ErhBMP-2 Inc. CPC preparation processes are outlined as follows:

First, amorphous calcium phosphate microparticles were coated onto CPC granules, mimicking body fluid precipitation under a 5-fold concentration for 24 hours at 37°C. These microparticles were then immersed in a supersaturated calcium and phosphorus solution for 48 hours at the same temperature, promoting the formation of crystalline calcium phosphate on their surfaces. After air-drying at room temperature, this precipitation cycle was repeated 3 times using the same 5-fold body fluid and supersaturated solution. During the third crystallisation, BMP-2 was incorporated into the solution, resulting in ErhBMP-2 Inc. CPC through co-deposition of BMP-2 and crystalline calcium phosphate.

### Created a beagle dog model for chronic horizontal alveolar bone defect

The study involved the participation of 5 healthy male beagles, each weighing between 15 and 20 kilograms and aged 18 to 24 months. The sample size (n = 5 per group) was determined based on prior experience with large-animal implant models and on commonly reported group sizes in comparable canine GBR studies. Throughout the study, the dogs were housed individually and maintained on a soft diet. From the initiation of therapy until study completion, plaque control was performed with twice-weekly brushing and daily rinsing with 0.5% chlorhexidine gluconate.

**Fig. 1:**
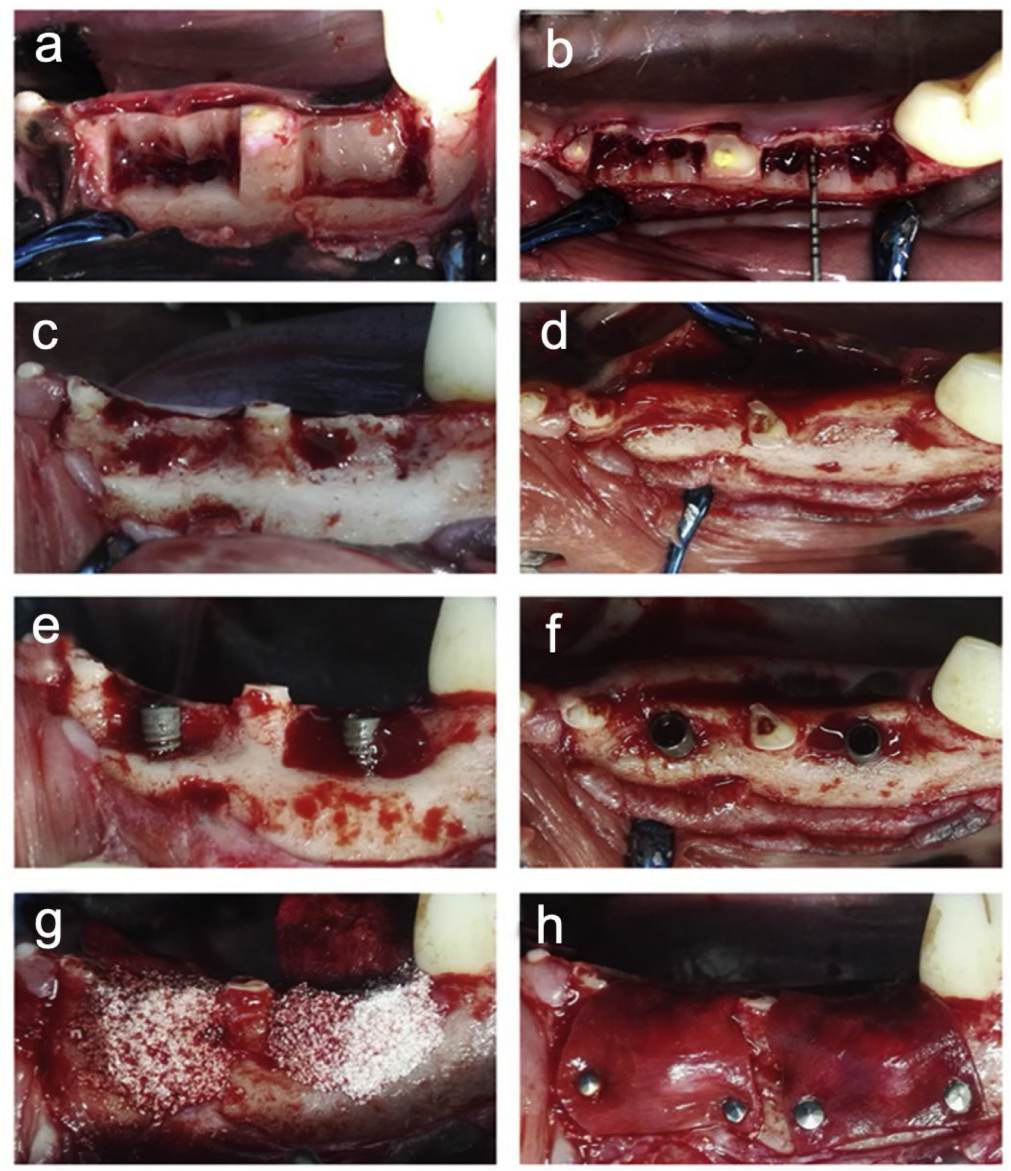
The process of surgery. A, Surgical removal the alveolar jawbone of the buccal side and the adjacent root space bone wall, with the lingual bone wall remaining intact. B and C, A bone defect was crafted. D, Three months later, the successful construction of a chronic alveolar bone defect model in the dog was achieved. E and F, Two implants were inserted. G, The GBR surgery was simultaneously conducted using randomised bone graft materials. H, A resorbable collagen membrane was utilised to cover the wound, securing it in place with pins. GBR, guided bone regeneration.

The procedure began with the administration of general and local anaesthesia to the beagle dogs. The distal root of the second premolar, the mesial root of the third premolar, and both roots of the fourth premolar in the mandible were surgically extracted following root separation. After root canal treatment of the mesial root of the second premolar and the distal root of the third premolar, the crowns of both teeth were trimmed to the alveolar bone level. A mucoperiosteal flap was then elevated to expose and remove the buccal alveolar bone extending from the distal root of the second premolar to the mesial root of the third premolar, as well as the buccal wall and root furcation bone of the fourth premolar, leaving the lingual wall intact. Four standardised defects were created per dog, each measuring 10 mm (mesio-distal) by 8 mm (bucco-lingual) by 5 mm (crown-root). After a 3-month healing period, chronic alveolar bone defect models were successfully established (Figures 1A–D).

### The procedure of simultaneous guided bone regeneration (GBR) and implant placement

Under general and local anaesthesia, a Bone Level Tapered (BLT) implant (Ø3.3 mm × 8 mm; Straumann, Basel, Switzerland) was placed at the centre of each bone defect, ensuring the implant’s lingual surface was completely covered by the bone wall. The buccal surface remained partially exposed, and the extent of exposure was recorded.

Each dog received 4 implants, and GBR was simultaneously performed using randomised grafting materials: autologous bone, CPC, high-dosage ErhBMP-2 (1088 µg/g) adsorbed onto CPC (ErhBMP-2 Ads. CPC), and low-dosage ErhBMP-2 (239 µg/g) incorporated into CPC via a biomimetic coating method (ErhBMP-2 Inc. CPC). The same grafting material was not used more than once per dog.

Finally, the surgical sites were covered with a resorbable collagen membrane (Bio-Gide, Geistlich Biomaterials) and sutured for closure (Figures 1E–H). All subsequent measurements and analyses were performed by investigators who were blinded to the group allocation. Appropriate analgesic protocols were followed for all surgical procedures, and animals were monitored daily for signs of pain, infection, or distress to ensure welfare throughout the study period.

### Radiographic observation and analysis

**Fig. 2:**
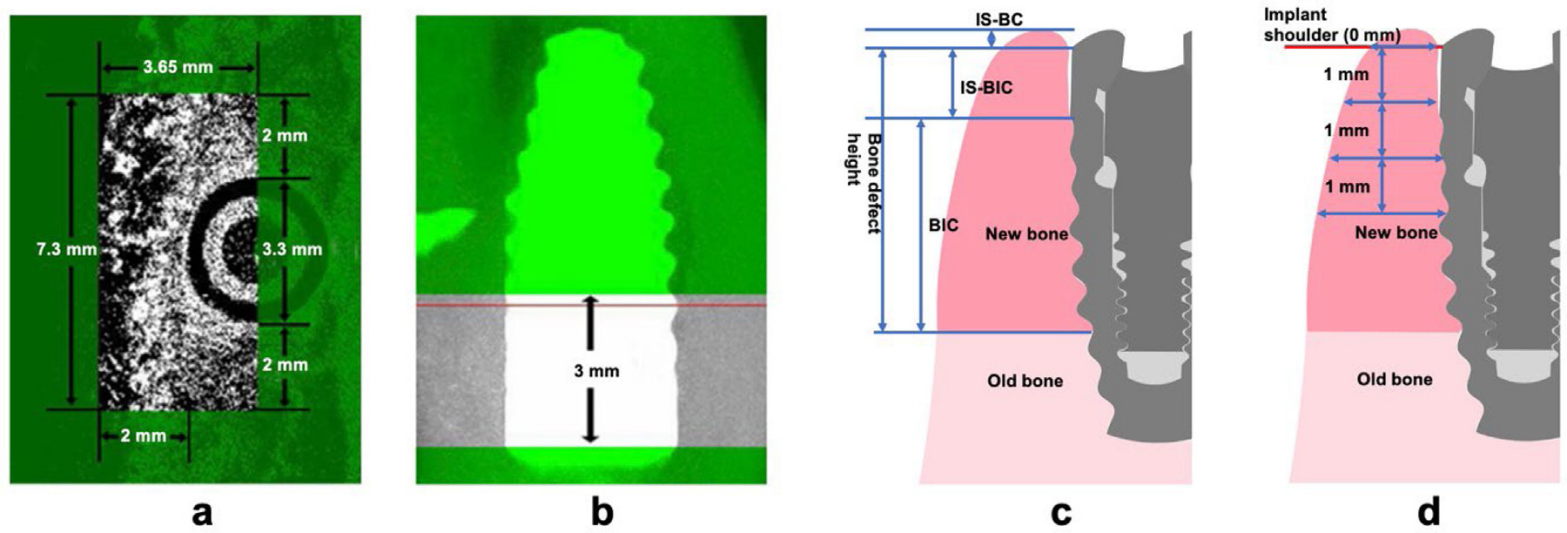
Measurement methods used in radiographic analysis and histomorphometric analysis. A and B, In the radiographic analysis, the VOI dimensions on the buccal side of the implant were 7.3 mm by 3.65 mm. C and D, The linear measurements for the histomorphometric analysis are shown in the vertical and horizontal directions. VOI, volume of interest.

Following a 3-month postimplant surgery period, the dogs were humanely euthanised. Tissue blocks were meticulously dissected and gathered for subsequent micro-CT scanning and histological analysis. They were removed, rinsed, and fixed in 10% buffered formalin solution. After 3 days of formalin fixation, the blocks underwent an overnight rinse in cold tap water. Subsequently, the blocks were scanned using a micro‐CT imaging system (SkyScan1176, Bruker micro‐CT), with parameters set at a voltage of 55 kV, a current of 114 mA, and a resolution of 15 µm per pixel. To reduce artifacts, a Gaussian filter (with a sigma value of 0.8 and support of 1) was applied. The volume of interest (VOI) was defined as 7.3 mm (mesio-distal) by 3.65 mm (bucco-lingual) by 3 mm (crown-root) (Figures 2A and B). The rationale for selecting the VOI dimensions (7.3 × 3.65 × 3 mm) was based on the critical role of peri-implant bone within 2 mm around the implant and the coronal 3 mm height of the implant neck in maintaining long-term implant stability. Therefore, the VOI was defined to include a 2 mm circumferential zone around the implant (implant diameter: 3.3 mm; radius: 1.63 mm) in the buccal, mesiodistal directions, and a vertical height of 3 mm from the implant neck region. And each group’s parameter was recorded after the samples were segmented for 3‐dimensional reconstruction, including:

1. bone mineral density (BMD).
2. bone volume fraction (BV/TV [%]).
3. Trabecula thickness (Tb. Th [µm]).
4. Trabecula number (Tb. N [1/µm]).
5. Trabecula separation (Tb. Sp [1/µm]).

### Histomorphometric analysis

The blocks were prepared for histomorphometric analysis by dehydration in ethanol and embedding in methylmethacrylate. Each block was sectioned buccolingually at 1000 µm intervals, then polished to a uniform thickness of 50 µm and mounted on plexiglass holders. Sections were stained with Basic Fuchsine, Toluidine Blue O, and McNeal's Tetrachrome. For analysis, 3 sections per implant were selected and independently evaluated by 2 experienced, blinded examiners. Assessments were performed using a Leica DC 200 digital camera with Leica QWin software (Leica Microsystems) and Image-Pro Plus (Media Cybernetics). Each parameter in every section was measured twice by both examiners. The parameters specific to the buccal side of the implant are as follows:

Linear measurements in the vertical direction (Figure 2C):

1. Bone defect height (mm): The vertical distance between the implant shoulder and the newly formed bone.
2. bone-to-implant contact, percentage of the implant boundary in contact with the newly formed bone (BIC [%]).
3. Distance between the implant shoulder to the most-coronal point of bone crest (IS-BC [mm]).
4. Distance between the implant shoulder to the most-coronal point of BIC (IS-BIC [mm]).

Linear measurements in the horizontal direction (Figure 2D):

New bone thickness at 0, 1, 2, and 3 mm below the implant shoulder.

Tissue components (in percent) within the augmented area:

1. New bone area percentage (NBA).
2. Residual material area percentage (RMA).
3. Nonmineralised area percentage (NMA).
4. Bone marrow area percentage (BMA).

### Statistical analysis

All statistical values were presented as mean ± standard deviation (SD), and statistical analysis was carried out with SPSS software (SPSS 16, IBM). Cronbach’s alpha (α) was calculated to confirm interrater reliability. Two-tailed tests were used for statistical significance testing, and *P* value less than or equal to .05 were considered statistically significant. The significance of differences in all groups was analysed using single-factor ANOVA, and Tukey's test was performed post-hoc multiple comparisons between groups.

## Results

### Clinical observations

Throughout the study period, it is noteworthy that all dogs maintained a state of good health. All implants exhibited osseointegration. Wound healing did not get impeded and was processed uneventfully. Importantly, no signs of pronounced inflammation were observed during the entire healing phase. No ectopic bone formation, abnormal inflammatory response, or wound healing complications were observed in any group during the experimental period.

### Radiographic observation and analysis

#### Radiographic observation

**Fig. 3:**
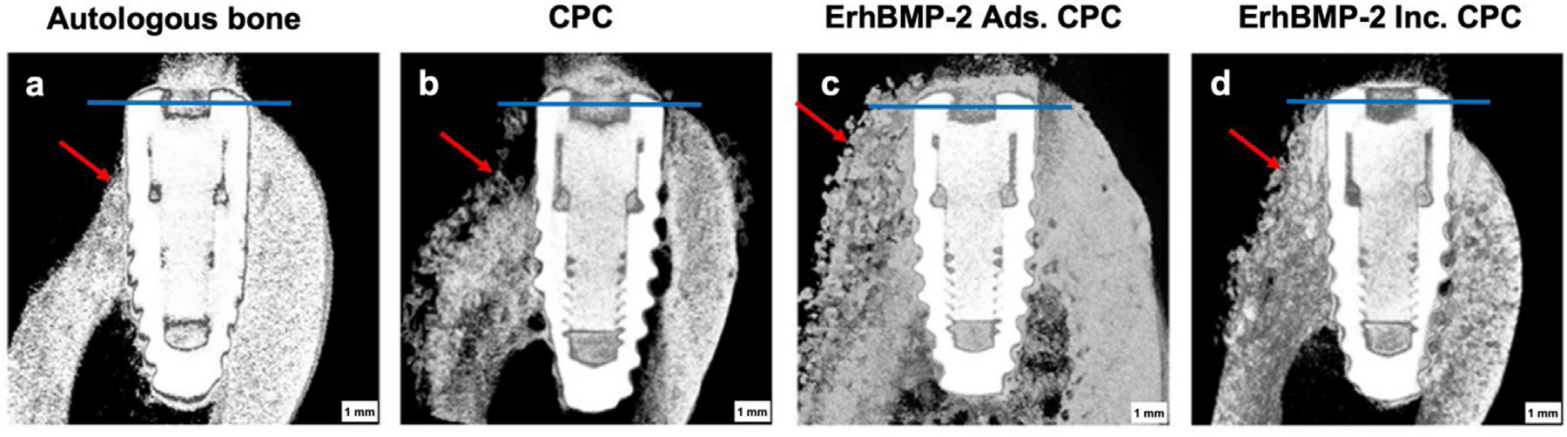
A-D, Micro-CT analysis of 3-dimensional reconstructed images in the 4 groups. Blue line: the implant shoulder. Red arrow: the augmented buccal bone.

At the twelve-week mark following the simultaneous GBR and implant placement surgery, the micro-CT-based 3-dimensional reconstructed images displayed a notable degree of variability within the 4 groups. In autologous bone and CPC groups, there was little radiographic evidence of bone formation on the buccal side of the implant neck, and the bone graft material was absorbed seriously. In contrast, the ErhBMP-2 Ads. CPC and ErhBMP-2 Inc. CPC groups exhibited the presence of multiple new bone formations along with residual grafting materials, clearly shown on the buccal side of the implant neck. Furthermore, these hard tissues displayed distinctive compositions. Notably, the ErhBMP-2 Inc. CPC group exhibited a more moderately mineralised bone structural tissue compared to the other groups (Figure 3).

#### Radiographic analysis

Based on the centre of the implant, the volume of interest (VOI) was defined as 7.3 mm (mesio-distal) by 3.65 mm (bucco-lingual) by 3 mm (crown-root) (Figure 2).

**Fig. 4:**
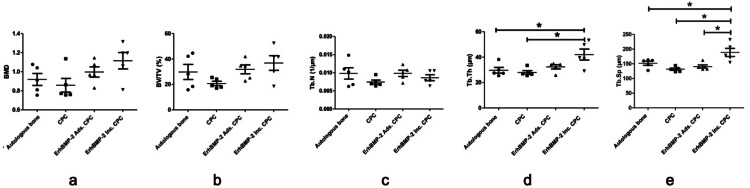
Quantification of radiographic analysis in 4 groups. A, The bone mineral density (BMD) analysis. B, The bone volume fraction (BV/TV) analysis. C, The trabecula number (Tb. N) analysis. D, The trabecula thickness (Tb. Th) analysis. E, The trabecular separation (Tb. Sp) analysis (n = 5, *P < .05).

The radiographic analysis results, presented in Figure 4, reveal essential findings. In the evaluation of bone mineral density (BMD), bone volume fraction (BV/TV), and Trabecula number (Tb. N), no significant differences were observed among all the groups under examination. However, a notable distinction emerged when considering trabecula thickness (Tb. Th). In this regard, the ErhBMP-2 Inc. CPC group (42.03 ± 9.648) exhibited a significantly higher value in comparison to the autologous bone group (29.69 ± 5.032) and the CPC group (27.98 ± 3.163). This observation underscores a noteworthy difference in trabecular thickness within these specific groups.

### Histological observations and histomorphometric analysis

#### Histological observations

**Fig. 5:**
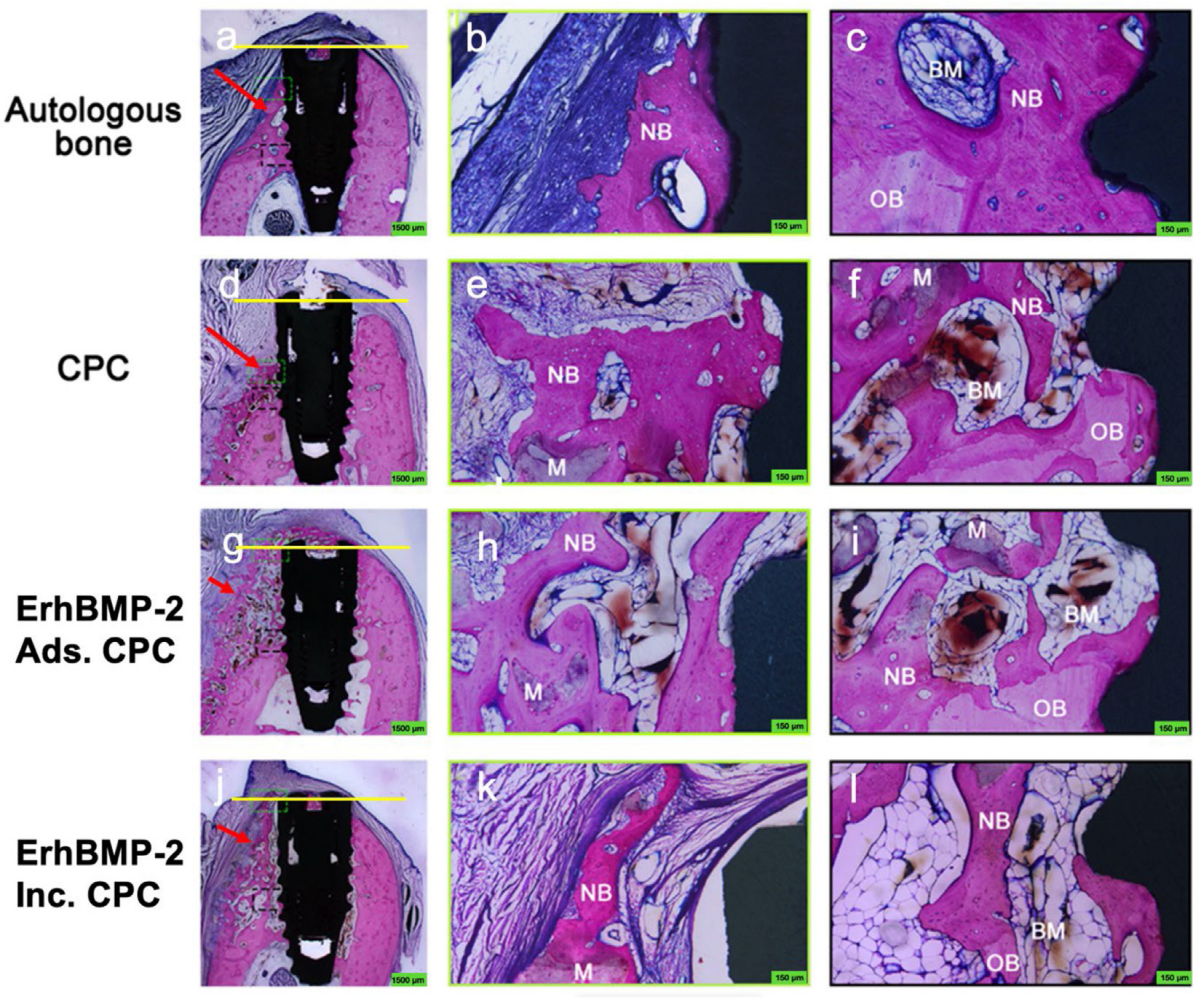
Histological evaluation of peri-implant bone regeneration at twelve weeks. Representative undecalcified sections from the 4 groups were stained with basic fuchsine, Toluidine Blue O, and McNeal's Tetrachrome to assess new bone formation. A-C, In the autologous bone group, continuous new bone formation was observed along the implant surface, particularly at the most coronal region on the buccal side. D-F, In contrast, limited newly formed bone was detected at the buccal implant neck in the CPC group, where fibrous tissue infiltration and incomplete osseointegration were evident. Enhanced peri-implant bone formation was observed in both ErhBMP-2 Ads. G-L, CPC groups, with more pronounced bone bridging and improved integration at the implant–bone interface, particularly in the ErhBMP-2 Inc. CPC group. In images A, D, G, and J, green dotted boxes indicate the most coronal region of the buccal bone crest, which is shown at higher magnification in B, E, H, and K. Black dotted boxes highlight the junction between pre-existing and newly formed bone, with corresponding magnified views in C, F, I, and L. BM, bone marrow; CPC, calcium phosphate cement; M, remained material; NB, new bone; OB, old bone. Yellow line: the implant shoulder. Red arrow: the augmented buccal bone.

In all groups, the presence of newly formed bone was evident on the buccal side of the implant. However, it's noteworthy that, in the autologous bone and CPC groups, there was minimal to no newly formed bone covering the neck of the implant's buccal surface. Comparatively, the ErhBMP-2 Inc. CPC group displayed a more advanced stage of newly formed trabecular bone development and a reduced presence of residual material when compared with the ErhBMP-2 Ads. CPC group. This observation underscores the enhanced maturity of the newly formed trabecular bone in the ErhBMP-2 Inc. CPC group (Figure 5).

#### Histomorphometric analysis

##### Linear measurements in the vertical direction

**Fig. 6:**
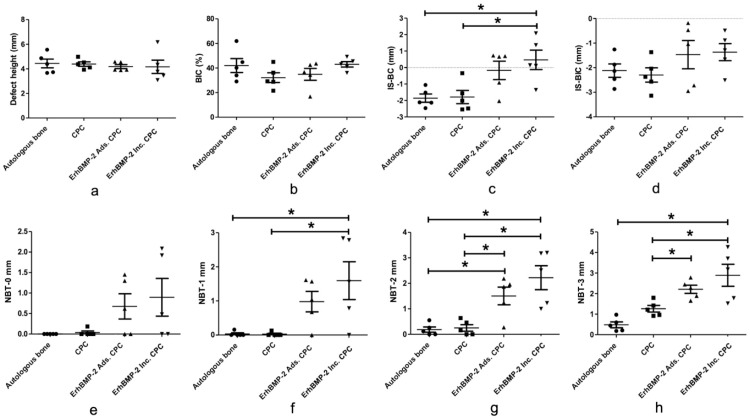
Quantification of the linear measurements in the vertical and horizontal direction across 4 groups. A, Defect height analysis. B, Bone-to-implant contact (BIC [%]) analysis. C, Distance between the implant shoulder to the most-coronal point of bone crest (IS-BC [mm]) analysis. D, Distance between the implant shoulder to the most-coronal point of BIC (IS-BIC [mm]) analysis. E-H, New bone thickness analysis at 0, 1, 2, and 3 mm below the implant shoulder (n = 5, *P < .05).

The results of linear measurements in the vertical direction are presented in Figures 6A-D. Notably, no statistically significant differences were observed in the measurements of defect height (mm), BIC (%), and IS-BIC (mm) in all groups. However, a notable distinction emerged when assessing IS-BC. Specifically, the IS-BC (mm) evaluation within the ErhBMP-2 Inc. CPC group (0.4667 ± 1.32) displayed a significant increase in comparison to both the autologous bone group (−1.857 ± 0.5621) and the CPC group (−1.789 ± 0.8997).

##### Linear measurements in the horizontal direction

The linear measurements in the horizontal direction results are shown in Figures 6E-H. Notably, the thickness of newly formed bone (NBT) at 0, 1, 2, and 3 mm below the implant shoulder exhibited considerable variability within all groups. At 0 mm below the implant shoulder, no statistically significant differences in NBT were observed across all the groups. However, when assessing NBT at 1, 2, and 3 mm below the implant shoulder, notable differences emerged. In the ErhBMP-2 Inc. CPC group, NBT demonstrated a significant increase compared to both the autologous bone and CPC groups. Furthermore, within the ErhBMP-2 Ads. CPC group, significantly higher NBT was observed at 2 mm and 3 mm below the implant shoulder in comparison to the CPC group, and NBT at 2 mm below the implant shoulder also exhibited a significant increase when compared to the autologous bone group. These findings underscore noteworthy variations in the thickness of newly formed bone in these specific locations.

##### Histomorphometric analysis of tissue components (in percent)

**Fig. 7:**
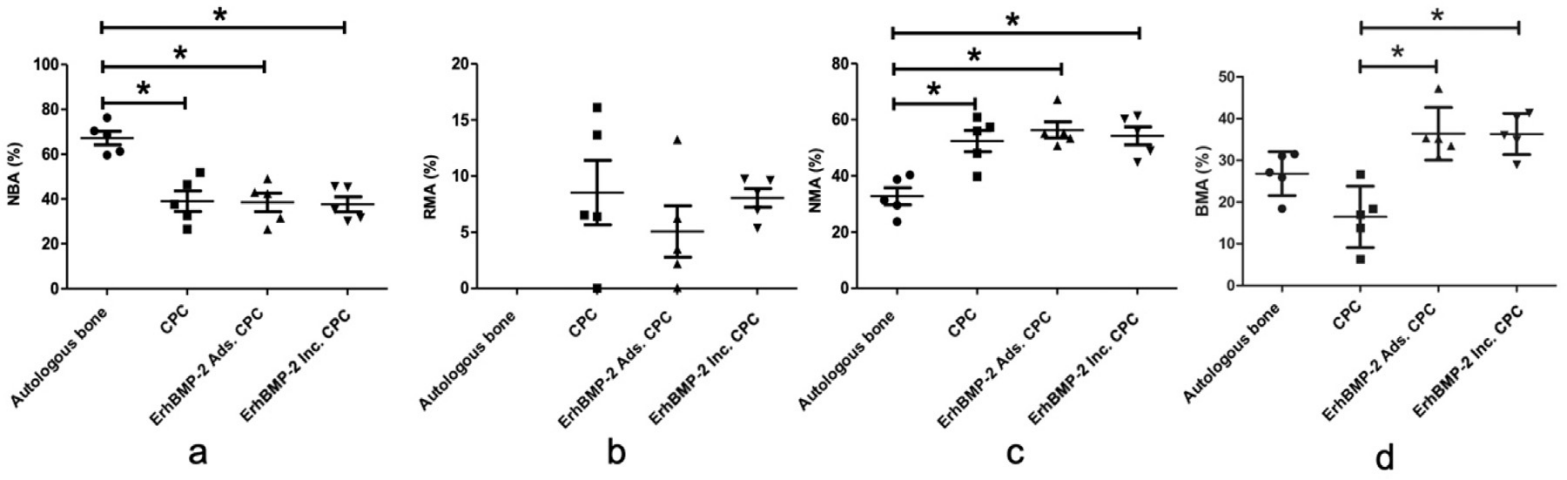
Quantification of tissue components (in percent) within the augmented area in 4 groups. A, New bone area percentage (NBA) analysis. B, Residual material area percentage (RMA) analysis. C, Nonmineralised area percentage (NMA) analysis. D, Bone marrow area percentage (BMA) analysis (n = 5, *P < .05).

Histomorphometric analysis of tissue components (in percent) within the augmented area revealed that there were no statistically significant differences in the residual material area (RMA) across all groups. However, important distinctions were observed in other parameters. Specifically, the percentage of new bone area (NBA [%]) in the autologous bone group was notably higher than in the other groups. Conversely, the percentage of nonmineralised area (NMA [%]) in the autologous bone group was significantly lower in comparison to the other groups (Figure 7).

## Discussion

Lateral ridge augmentation presents a more challenging regenerative condition than socket preservation or vertical augmentation models. Horizontal defects are typically noncontained and lack sufficient bony walls to confine graft materials, making volume maintenance and contour stability more difficult under soft-tissue pressure. When augmentation is performed simultaneously with implant placement, additional complexity arises, as primary implant stability must be achieved while bone regeneration is still developing. Micromotion at the graft–implant interface and the need for coordinated osseointegration and graft remodelling further distinguish this scenario from previously studied models. In the present study, ErhBMP-2 Inc. CPC achieved regenerative outcomes comparable to those of a higher adsorption dose. This observation suggests that sustained local release, rather than absolute dose, may be more relevant in maintaining effective osteogenic signalling in this biologically demanding environment.

Based on the radiographic and histological results, ErhBMP-2 Inc. CPC, as a novel bone substitute for simultaneous lateral ridge augmentation with implant placement, demonstrated superior bone regeneration and effective alveolar contour preservation compared to CPC alone at twelve-week postsurgery. These findings confirm the osteoinductive potential of ErhBMP-2 in GBR, consistent with previous studies.^30^, ^31^, ^32^, ^33^ In Wikesjö’s 2002 study, an organic bone substitute material (αBSM) served as the rhBMP-2 carrier, with the growth factor solution injected into αBSM powder. In 2004, an absorbable collagen sponge (ACS) was instead used as the carrier. In contrast, the present study utilised ErhBMP-2 Inc. CPC, enabling localised, controlled, and sustained BMP-2 release through a cell-mediated mechanism that closely mimics natural bone remodeling.^22^^,^^23^ However, release kinetics were not directly measured; therefore, the improved performance observed here should be interpreted as functional outcomes rather than as direct evidence of a specific delivery mechanism.

In the current study, the ErhBMP-2 Inc. CPC group demonstrated significantly greater trabecular thickness (Tb. Th) radiographically and higher histomorphometric values than the CPC group. This is attributed to BMP-2′s osteoinductive properties, which impart osteoinductivity to CPC irrespective of delivery system or concentration, aligning with Olthof et al.^34^ Furthermore, BMP-2 accelerates bone maturation, leading to thicker trabeculae earlier, a result consistent with Lee et al.,^35^ who reported greater Tb. Th in rhBMP-2 group at 3 weeks.

In the present study, although the BMP-2 concentration in the ErhBMP-2 Inc. CPC group was only one-tenth of that in the ErhBMP-2 Ads. CPC group, no significant differences were observed in peri-implant bone regeneration or ridge contour preservation. This finding suggests that increasing the total BMP-2 dose beyond a certain threshold may not proportionally enhance bone formation. One possible explanation is that the osteogenic response to BMP-2 reaches a plateau once local receptor-mediated signalling pathways are sufficiently activated. Moreover, the sustained-release characteristics of the ErhBMP-2 Inc. CPC system may improve local bioavailability and maintain biologically effective concentrations over time, thereby reducing the need for higher initial doses. This interpretation is consistent with previous findings demonstrating that controlled and prolonged BMP-2 delivery enhances osteogenic efficiency compared with burst-release systems.^36^ From a translational perspective, achieving comparable regenerative outcomes with a lower BMP-2 dose may also help mitigate potential dose-related adverse effects.

The reconstruction and preservation of alveolar contours around the implants in the ErhBMP-2 Inc. CPC group showed notable outcomes in both vertical and horizontal dimensions compared with the autologous bone group. However, bone regeneration efficacy was slightly lower than that achieved with autologous bone. These findings differ from our previous study,^26^ which reported no significant differences in new bone formation or alveolar contour preservation between the 2 groups. This discrepancy may be attributed to the use of autologous bone granules in the present study, which have limited ability to maintain alveolar shape and resorb faster than other substitutes. In contrast, the earlier study employed denser autologous bone blocks—facilitating vertical ridge augmentation and exhibiting slower biodegradation than granules.

This study has several limitations. First, a blank control group without GBR was not included. The experimental model involved chronic horizontal alveolar defects in beagle dogs, which are not expected to heal spontaneously without grafting intervention. For this reason, inclusion of an untreated control was considered neither ethically justified nor scientifically informative for the specific aims of this study. Second, although no macroscopic abnormalities were noted in major organs at the time of tissue harvesting, comprehensive systemic safety assessments, including haematological or histopathological analyses, were not performed. Therefore, the systemic safety profile of ErhBMP-2 delivery warrants further investigation. In addition, BMP-2 release kinetics and downstream biological signalling pathways were not directly examined. Mechanistic interpretations regarding sustained release or enhanced bioavailability are therefore based on indirect histological findings and previously published evidence rather than direct molecular measurements. Considering the known differences in BMP-2 dosing between animal studies and human applications, establishing an appropriate clinical dose should be the first priority in future translation. The concentrations used in preclinical models are often higher than those considered acceptable in patients, and excessive BMP-2 exposure has been linked to adverse reactions, including abnormal bone formation and local inflammatory responses. Therefore, early-phase clinical trials should focus on identifying the lowest effective dose that can achieve predictable bone regeneration while maintaining safety. Further optimisation of delivery systems may also help reduce total dosage requirements and improve local control of BMP-2 activity.

## Conclusion

This study demonstrated that ErhBMP-2 Inc. CPC enhanced peri-implant new bone formation and better-preserved alveolar ridge contours compared with CPC alone during simultaneous lateral ridge augmentation with implant placement in a canine model over a 12-week period.

## Author contributions

Concept and design: Yuelian, Zhonghao, Yiqun.

Acquisition, analysis, or interpretation of data: All authors.

Drafting of the manuscript: All authors.

Critical revision of the manuscript for important intellectual content: All authors.

Statistical analysis: Lingfei, Yuanyuan.

Administrative, technical, or material support: Yuelian, Zhonghao, Yiqun.

Supervision: Yuelian, Zhonghao, Yiqun.

Final approval and accountability: All authors.

## Conflict of interest

The authors declare no other potential conflicts of interest with respect to the research, authorship, and/or publication of this article.
